# Photoprotection Against UV-Induced Skin Damage Using Hyaluronic Acid Produced by *Lactiplantibacillus plantarum* and *Enterococcus durans*

**DOI:** 10.1007/s00284-023-03377-y

**Published:** 2023-06-27

**Authors:** Amany E. Shaheen, Hassan M. Gebreel, Loutfy A. Moussa, Abeer E. Zakaria, Waleed A. Nemr

**Affiliations:** 1 Department of Radiation Microbiology, National Center for Radiation Research and Technology (NCRRT), Egyptian Atomic Energy Authority (EAEA), Cairo, Egypt; 2 Department of Microbiology, Faculty of Science, Ain Shams University, Cairo, Egypt

## Abstract

Exposure to ultraviolet (UV) radiation is one of the major factors that causes skin aging, erythema, sunburns, and skin cancer. This study aimed to select probiotic bacterial isolates able to produce high yield of hyaluronic acid (HA) to be employed for skin photoprotection and other possible biological applications. The selected isolates K11 and St3 were able to produce the highest yields of HA 4.8 and 4.4 mg/ml, respectively. Both isolates were identified as *Enterococcus durans* strain K11 and *Lactiplantibacillus plantarum* strain St3 using 16S rRNA gene sequencing. The antioxidant activity of HA produced by *E. durans* strain K11 and *L. plantarum* strain St3 was (65.4 0.2%) and (66.6 0.1%), respectively. The viability of UVB-irradiated keratinocytes pre-treated with HA produced by *E. durans* strain K11 and *L. plantarum* strain St3 was 91.3 and 91.4%, respectively, compared with the control. While the viability of UVB-irradiated keratinocytes post-treated with HA produced by *E. durans* strain K11 and *L. plantarum* strain St3 was 86 and 88.5%, respectively. To the best of our knowledge, this is the first recordation of HA production by *Enterococcus durans* and *Lactiplantibacillus plantarum* which revealed a significant radioprotection of the human keratinocytes against UVB radiation*.*

**Supplementary Information:**

The online version contains supplementary material available at 10.1007/s00284-023-03377-y.

## Introduction

Major health problems linked to overexposure to UV radiation, such as skin cancer (melanoma and non-melanoma), premature aging of the skin, actinic keratoses, sunburns, cataracts, pterygium, degeneration of the macula, and suppression of body’s immune system and the skin’s natural defenses [1].

Oxidative stress, brought on by prolonged exposure to UV radiation from sunlight, usually affects human skin, can lead to cellular damage (e.g., lipid peroxidation and DNA fragmentation), apoptosis, and cell death. Skin overexposed to UV is linked to a variety of abnormal responses, such as inflammatory responses, epidermal hyperplasia, the breakdown of collagen, and the formation of melanin [2]. According to the wavelength, UV can be classified into the three subtypes of UVA, UVB, and UVC. In recent years, more attention has been paid to the increased UVB radiation that is now reaching the Earth’s surface because of the loss of stratospheric ozone. Unlike the other two subtypes of UV, UVB has a medium wavelength and is known to cause higher cellular stress in humans and harm the ecosystem. UVB is known to affect human health by promoting the production of reactive oxygen species (ROS) [3]. Cellular DNA is susceptible to UVB damage, either directly through DNA absorption of mutagenic radiations, which causes lesions known as the UV “signature” to increase (pyrimidine dimers or 6–4 photoproducts) or indirectly through interaction with other biochromes forming reactive oxygen species (ROS) which have negative effects on cells and can enter the nucleus where they can cause oxidative DNA alterations and strand breaks [4]. Skin photoprotection is necessary to protect skin from the harmful effect of UV. This includes wearing clothing, sunglasses, and sunscreens. Modern topical skin photoprotection includes both primary protective factors (sunscreens) that absorb or reflect UV radiation and secondary protective factors (such as antioxidants, osmolytes, and DNA repair enzymes) that can interfere with the photochemical reactions generated by UV penetration, thereby reducing skin damage [5].

Oral and topical probiotics have the potential to prevent and treat skin photoaging by altering the skin microbiome and gut–skin microbial interactions. These mechanisms include lowering oxidative stress, preventing extracellular matrix remodeling, reducing inflammatory cascade reactions, and maintaining immune homeostasis [6]. A few research have concentrated on the use of *L. plantarum* extracts in skin care, even though bacteria are assumed to play a significant influence in the prevalence of acne, skin hydration, and nutrient metabolism [7]. There has been evidence of the health benefits of some specific probiotic strains, including *Bifidobacterium, Saccharomyces, Enterococcus, Bacillus, and Lactobacillus* [8]. Also, research revealed that *L. plantarum* protects hairless mice and human skin fibroblasts from UVB-induced photoaging [9].

Hyaluronic acid, also known as hyaluronan, is a linear polysaccharide which is one of the main components of connective tissue, forming a gelatinous environment that surrounds cells. HA is made up of repeated units of (1,4)-D-glucuronic acid (GlcUA) and (1,3)-N-acetylglucosamine (GlcNAc), which are connected alternately by glycosidic linkages. In humans, HA is found in all body tissues and fluids, but it is most prevalent in the skin (which accounts for 50% of the body’s total HA), the vitreous of the eye, the umbilical cord and synovial fluid. It is also found in skeletal tissues, heart valves, the lung, the aorta, the prostate, tunica albuginea, corpora cavernosa, and the corpus spongiosum of the penis. Mesenchymal cells produce the majority of HA; however, other cell types can as well [10].

Commercial hyaluronan is mainly produced by biotechnology (microbial fermentation). HA generated from microorganisms is compatible with the human body [10]. Conventionally, *Streptococcus* species have been employed to produce HA at industrial scale. *S. zooepidemicus* is the most broadly applied strain in the production of HA. Many studies have revealed that certain strains of lactobacilli and bifidobacteria are able to produce hyaluronic acid, which could improve the barrier, immunological, and homeostatic functions of the skin [11–13].

The antioxidant activity of hyaluronic acid in the body as an effective free radical scavenger is one of its most important functions [14]. Hyaluronic acid showed strong inhibition of lipid peroxidation and scavenging activities of hydroxyl radical. Hyaluronic acid can neutralize free radicals, which is also known to provide protection [15]. Hyaluronic acid contributes to the maintenance of stronger skin and shielding it from the damaging effects of UV radiation. The antioxidant properties of HA also help reducing the damaging effects of UV on the skin [16].

In the current study, *Enterococcus durans* and *Lactiplantibacillus plantarum* were reported as HA producers for the first time. The extracted HA showed an antioxidant activity and good protective effect to the human dermal keratinocytes against UVB damage.

## Material and Methods

### Isolation of the Probiotic Bacteria

Three different samples, kefir (K), breast milk 1 (H), and breast milk 2 (Z), were collected from different sources. Kefir sample was purchased from Healthy Corner, Heliopolis, Cairo (30°05′24.8″ N 31°19′06.4″ E). Two breast milk samples were collected from two different newly delivered women. From each sample, 1 ml was serially diluted in phosphate-buffered saline solution (PBS) (pH ~ 7.4) then, separately inoculated into three different agar media: deMan Rogosa and Sharpe (MRS) [17], Kanamycin Aesculin Azide [18], and KF streptococcus agar [19] by pour plate method. The plates were aerobically incubated at 37 °C for 24 h.

In addition to the isolated cultures four probiotic strains (*Lactiplantibacillus plantarum* strain M and *Lactiplantibacillus plantarum* strain St3) were isolated by Shaheen [20], and (*Lactobacillus casei* NRRL-1922 and *Lactobacillus acidophilus* NRRL-23431) were kindly supplied from Agricultural Research Services Culture Collection, Peoria, United States.

### Extraction of Hyaluronic Acid from the Selected Cultures

The pure cultures isolated from kefir as well as breast milk samples were tested for their ability to produce hyaluronic acid by cultivating them aerobically in 10% skimmed milk medium and then incubated at 37 °C for 24 h with no agitation. The fermented broth was centrifuged at 10,000 g for 15 min at 25 °C. The supernatant was collected for qualitative and quantitative determination of hyaluronic acid [11].

Proteins were removed using trichloroacetic acid 4% (w/v) then incubation for 1 h at 4 °C, followed by centrifugation for 20 min at 10,000 g at 4 °C [21]. One volume of the supernatant was mixed with 2.5 volumes of chilled absolute ethanol and incubated at 4 °C for 1 h. The samples were then centrifuged at 10,000 g for 15 min at 4 °C. The pellet was dissolved in five volumes of deionized water [11, 12].

### Turbidimetric Analysis of Hyaluronic Acid

According to the method reported by Hor et al. [12] HA samples were analyzed using the cetyltrimethylammonium bromide (CTAB) turbidimetric method. CTAB reagent was prepared by dissolving 2.5 g of CTAB (Sigma, Aldrich) in 100 ml of 0.2-M NaCl solution. One milliliter of HA standard and samples were mixed gently with 2 ml of CTAB reagent and allowed to rest for 10 min before measuring the absorbance at 400 nm using UV–Vis spectrophotometer. HA concentration in samples is calculated based on a standard curve obtained using different HA concentrations (10–100 µg/ml).

### Analysis of the Extracted Hyaluronic Acid by HPLC

The concentration of the extracted HA from different cultures was measured by reversed phase (RP) HPLC according to Vigliano et al. [22] with some modifications. The HPLC analysis of hyaluronic acid was performed at National Center for Radiation Research and technology, Egyptian Atomic Energy Authority, Egypt, using Zorbax Agilent Eclipse plus RP C18 column (4.6 × 100 mm), mobile phase (60%) acetonitrile and (40%) water (isocratic elution). A flow rate was 1.0 ml/min and the sample injected volume was 5.0 µl. The UV detector was used to detect the output signals at 200 nm.

### Characterization of the Produced Hyaluronic Acid by FTIR

The extracted HA from different cultures of the selected bacterial isolates was compared with standard HA using FTIR spectroscopy [23]. FTIR analysis was performed at National Center for Radiation Research and technology, Egyptian Atomic Energy Authority, Egypt, by Vertex 70v FTIR Spectrometer (Bruker). All samples, extracted HA and standard HA, were dissolved in deionized water and directly measured. The absorption was measured between 400 and 4000 cm^−1^.

### Identification of the Selected Hyaluronic Acid Producing Isolate by 16S rRNA Sequencing

The PCR was carried out at Microbiology Department, National Center of Radiation Research and Technology (NCRRT), Atomic Energy Authority (AEA). Total DNA of the selected bacterial isolate showed the highest production of HA was extracted using DNA extraction kits (Thermo; Fisher Scientifics; USA) according to manufacturer’s instructions. PCR was performed using Premix *Taq* (Ex *Taq* Version, Takara, Japan) according to instruction manual. A pair of universal bacterial primers was used to partially amplify target 16S rRNA gene from the bacterial isolates 27F (5′-AGA GTT TGATCC TGG CTC AG-3′) and 1492R (5′-GGT TAC CTT GTT ACG ACT T- 3′). A portion of 2 μl of bacterial DNA was transferred into a 50-μl final volume as template for PCR. PCR was performed using Maxima Hot Start PCR Master Mix (Thermo K1051) according to manufacturer’s instructions. PCR was performed in genius model FGENO2TD thermal cycler (Techne, England). The PCR conditions were adjusted to 5 min for initial denaturation at 94 °C, then 35 cycles of 1 min at 94 °C, 1 min at 54 °C, and 1 min at 72 °C and then finally 10 min at 72 °C. The amplified genes were run on 1% agarose gel and visualized with standard marker of known size (ladder 100, Wako, Japan) to determine the size and purity of products and then amplified bands were cleaned using GeneJET™ PCR Purification Kit (Thermo K0701).

Sequencing of forward and reverse directions of PCR product of 16S rRNA gene was carried out by Macrogen, Korea. The obtained sequences were identified using BLAST search program, National Center for Biotechnology Information (NCBI), National Library of Medicine, USA [24] and the online EzBiocloud server (http:// www. ezbiocloud.net [25]. Sequence alignments were performed by MEGA V6 software [26] and then blasted on GenBank and all closely related species were downloaded to construct the phylogenetic tree using neighbor-joining methods. The sequences were submitted using Bankit tool (NCBI, website) to obtain the accession numbers.

### The Antioxidant Activity of the Produced Hyaluronic Acid

The scavenging effect of the produced HA on 2,2-diphenyl-1-picrylhydrazyl (DPPH) free radicals was evaluated. Two milliliters from each HA sample of concentration 100 µg/ml (the produced and the standard) were added to 0.1-mM DPPH dissolved in 95% ethanol. The mixture was shaken and left for 30 min at room temperature and the absorbance of the resulting solution was measured at 517 nm [20, 27]. A lower absorbance represents a higher DPPH radical-scavenging activity. Ascorbic acid (AA, 100 µg/ml) was used as a positive control. The percentage of scavenging effect was expressed as shown in the following equation:

$${\text{DPPH}}\, - \,{\text{scavenging}}\,{\text{activity}}\,\left( \% \right)\, = \,\left[ {\left( {{\text{A}}_{{{\text{control}}}} \,{-}\,{\text{A}}_{{{\text{sample}}}} } \right)/{\text{A}}_{{{\text{control}}}} } \right]\, \times \,100.$$

### The Radioprotective Effect and Cytotoxicity of the Produced Hyaluronic Acid

#### HA Preparation

Stock solutions (1 mg/ml) of the produced and commercial HA from L'Oréal (1.5%) were prepared in cell culture media Dulbecco’s modified Eagle’s medium (DMEM, Sigma, St Louis, Missouri) supplemented with 10% fetal bovine serum (FBS), glutamine (0.29 mg/ml), and gentamycin (50 µg/ml). The tested concentration of HA (1 mg/ml) was selected according to Hašová et al. [28].

#### Cell Culture Preparation

The study was applied on keratinocytes from foreskin in children. After circumcision, pediatric foreskin was transferred to the laboratory in DMEM at 4 °C. The skin was rinsed in phosphate-buffered saline with antibiotics and antimycotics. As much as 4 cm^2^ of tissue, by each foreskin, was harvested for double-enzymatic digestion. Subcutaneous fat was removed with scissors and the remaining tissue was cut into roughly 0.5 cm^2^ pieces and incubated in 20-ml Dispase for 12 h at 4 °C. After incubation the epidermis was lifted off the dermis with pliers and transferred into 5-ml trypsin. The tissue was incubated at 37 °C in 95% humidity and 5% CO_2_ for 15 min and during this time it was repeatedly removed from the incubator and triturated. After incubator and triturated, the suspension was centrifuged at 1400 rpm for 10 min then the supernatant was removed, and the pellet of human keratinocyte cells was resuspended in culture medium [29]. Human keratinocyte cells were grown in DMEM supplemented with 10%FBS, glutamine, and gentamycin in 5% CO_2_ at 37 °C [28].

#### UVB Irradiation

UVB irradiation was carried out by means of UVB broadband TL Philips lamp emits radiation in the B bandwidth of UV spectrum (290–315 nm) and the UV intensity was measured using Waldmann Variocontrol UV meter, Germany and then the dose was calculated. The irradiation dose (single dose 10 mJ/cm^2^) used in this experiment was selected according to Hašová et al. [28]. This experiment was carried out in 2 sets: pre-treatment and post-treatment. In case of pre-treatment, before UVB treatment, the cell culture media were replaced by cell culture media with or without HA and then were exposed to UVB irradiation (single dose 10 mJ/cm^2^). The cells and cell culture media were incubated and harvested 24 h after UVB irradiation. In case of post-treatment, immediately after the UVB irradiation (single dose 10 mJ/cm^2^), culture media was replaced by cell culture media with or without HA and then were incubated and harvested after 24 h.

#### Cell Viability Using MTT Assay

Cell viability was measured using the 3-(4,5-dimethylthiazol-2-yl)-2,5-diphenyltetrazolium bromide (MTT, Sigma-Aldrich) assay [30]. The optical density was measured at a wavelength of 570 nm [28].

### Statistical Analysis

All tests are performed in triplicates, and data were expressed as mean ± standard error (SE). Statistical significance was assessed using one-way ANOVA (analysis of variance, SPSS software v.18) test and the means were compared with Duncan’s test at 0.05 level.

## Results

### Isolation and Screening of the Selected Isolates for Hyaluronic Acid Production

**Fig. 1:**
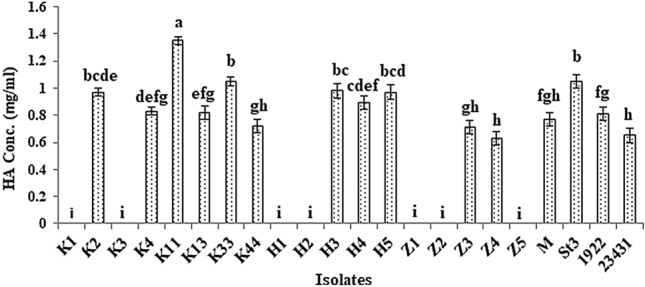
Concentrations of hyaluronic acid produced by the selected isolates using turbidimetric assay. Data are shown as the mean ± SE of triplicate measurements from independent experiments. Statistical significance was assessed using one-way ANOVA (analysis of variance, SPSS software v.18) test and the means were compared with Duncan’s test at 0.05 level. Accordingly, mean values with different small letters are considered statistically different (*P*≤0.05)

Eighteen bacterial isolates were selected from different sources grown on MRS agar, Kanamycin aesculin azide agar, and KF streptococcus agar media. In addition to the selected isolates, *L. plantarum* strain M*, L. plantarum* strain St3*, L. casei* 1922 and *L. acidophilus* 23,431 were examined to produce hyaluronic acid by turbidimetric method. Fifteen out of twenty-two bacterial isolates were able to produce hyaluronic acid (Fig. 1). The highest production of hyaluronic acid accomplished by K2, K11, K33, H3, H5 and St3 isolates was 0.97 ± 0.03, 1.35 ± 0.03, 1.05 ± 0.03, 0.98 ± 0.05, 0.97 ± 0.05, and 1.05 ± 0.05 mg/ml, respectively. There was a significant difference (at *P*≤0.05) in the concentration of HA produced by the isolate K11 and isolates St3 and K33. The highest HA production was recorded by the isolates K11, St3, and K33 compared with the other isolates.

### HPLC Analysis of the Produced Hyaluronic Acid

**Fig. 2:**
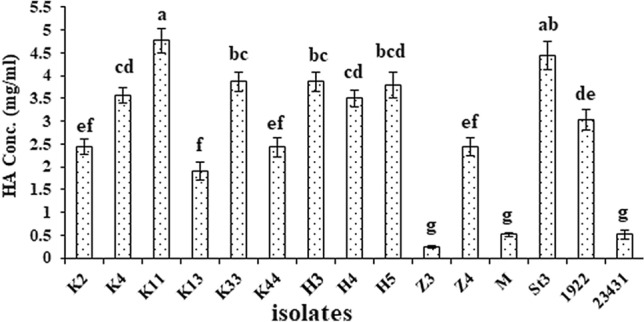
Hyaluronic acid productivity of the selected isolates by HPLC. Data are shown as the mean ± SE of triplicate measurements from independent experiments. Statistical significance was assessed using one-way ANOVA (analysis of variance, SPSS software v.18) test and the means were compared with Duncan’s test at 0.05 level. Accordingly, mean values with different small letters are considered statistically different (*P*≤0.05)

The fifteen HA producing bacterial isolates were analyzed by HPLC as represented in figure S1. The results of HPLC analysis confirmed the production of HA by all tested bacterial isolates at the same retention time compared with standard HA which was detected at retention time 1.089 min. Eight bacterial isolates (K4, K11, K33, H3, H4, H5, St3, and 1922) showed high HA productivity ranged from 3 to 4.8 mg/ml. The production of HA by K11 and St3 was significantly high (at *P*≤0.05) about 4.8 ± 0.3 and 4.4 ± 0.2 mg/ml, respectively (Fig. 2).

### Characterization of Hyaluronic Acid by FTIR

The FTIR spectra of the produced HA by different isolates and standard HA showed similar fingerprints (Fig. S2). The produced HA by the selected isolates showed characteristic peaks at 3322, 2173, 1638, 605, and 587 cm^−1^. The broad band at approximately 3329 cm^−1^ is attributed to hydrogen-bonded O–H. The amide I group of C = O carboxyl is ascribed to the peak at 1640 cm^−1^. Peak at 1043 cm^−1^ indicated the alcohol C–OH group. The peaks ranged from 603 to 642 cm^−1^ could be due to the C–O–C stretching.

### Identification of the Selected Probiotic Bacterial Isolates by 16S rRNA Gene

**Fig. 3:**
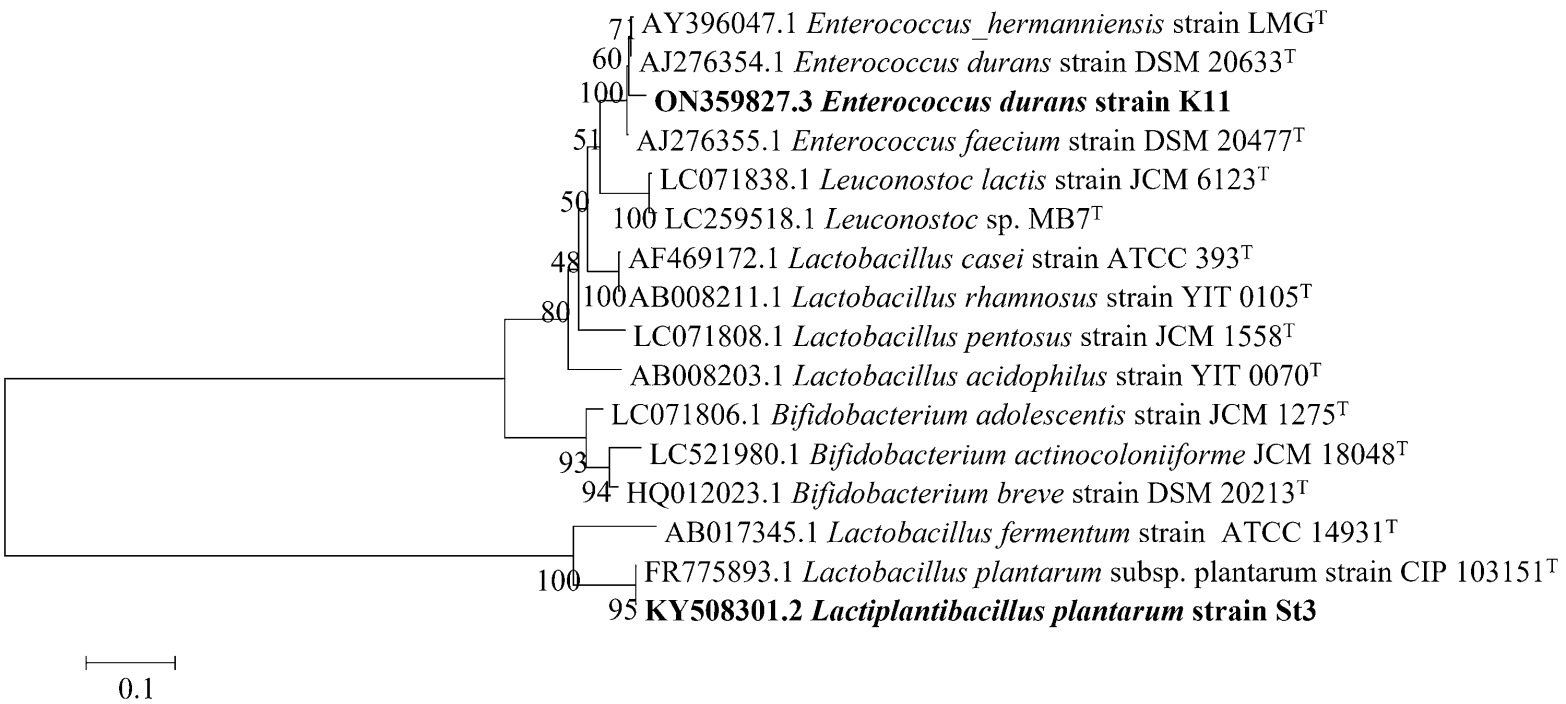
Neighbor-joining phylogenetic tree of 16S rRNA genes. The numbers at the nodes are bootstrap values recovered from 100 trees

The probiotic isolates (K11 and St3) were selected as the most promising HA producing isolates. The isolate St3 was isolated and identified using 16S rRNA gene by Shaheen [20] as *Lactiplantibacillus plantarum* strain St3 and recorded at the GenBank under the accession number (KY508301). Bacterial isolate K11 was identified using 16S rRNA gene as *Enterococcus durans*. The nucleotide sequences were submitted to GenBank under accession number (ON359827). Neighbor-joining phylogenetic tree with alignment of 16S rRNA gene revealed that *L. plantarum* strain St3 was placed in the phylogenetic cluster with *L. plantarum* strain CIP 103151 type strain, while *E. durans* strain K11 in the cluster with *E. durans* strain DSM 20633 type strain. The topology of the phylogenetic tree clearly confirmed the NCBI-BLAST results and the belonging of St3 and K11 strains to genus *Lactobacillus* and *Enterococcus*, respectively and thus identification of strain St3 as *L. plantarum* and strain K11 as *E. durans* (Fig. 3).

### Antioxidant Activity of the Produced Hyaluronic Acid

**Fig. 4:**
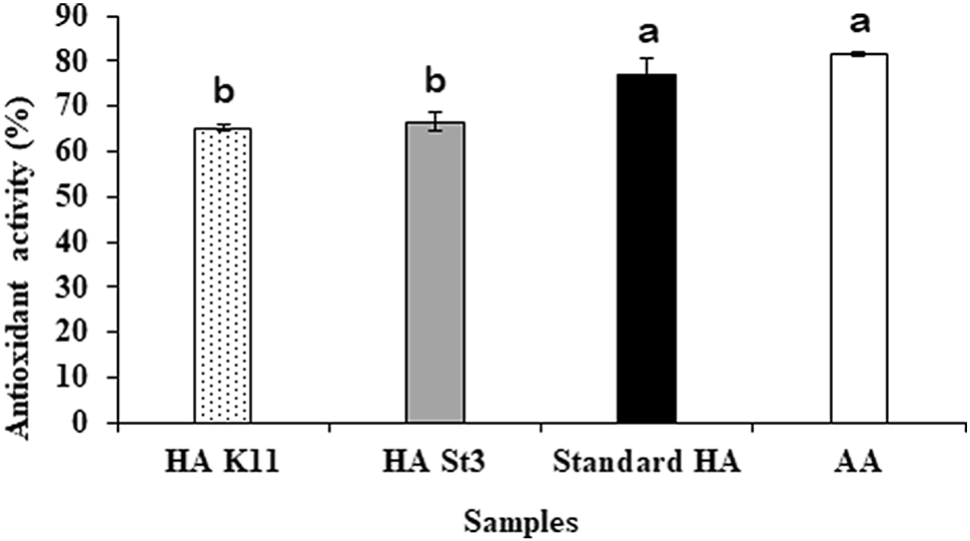
Antioxidant activity of the produced HA by *E. durans* strain K11 and *L. plantarum* strain St3. Data are shown as the mean ± SE of triplicate measurements from independent experiments. Statistical significance was assessed using one-way ANOVA (analysis of variance, SPSS software v.18) test and the means were compared with Duncan’s test at 0.05 level. Accordingly, mean values with different small letters are considered statistically different (*P*≤0.05)

The antioxidant activity of the produced HA was (65.4 ± 0.2%) and (66.6 ± 0.1%) by *E. durans* strain K11 and *L. plantarum* strain St3, respectively (Fig. 4). The results showed that the radical-scavenging capacity of ascorbic acid (positive control) and standard HA was significantly high (at *P*≤0.05) compared with that of the produced HA. Also, there is no significant difference of antioxidant activity between the produced HA by *E. durans* strain K11 and *L. plantarum* strain St3. The produced HA by *E. durans* strain K11 and *L. plantarum* strain St3 showed good antioxidant activity which is very close to that of ascorbic acid and standard HA. The variation in significance may be attributed to the difference in the parity between the highly purified standard HA and the extracted HA samples.

### The Radioprotective Effect of the Hyaluronic Acid Produced by E. durans Strain K11 and L. plantarum Strain St3

**Fig. 5:**
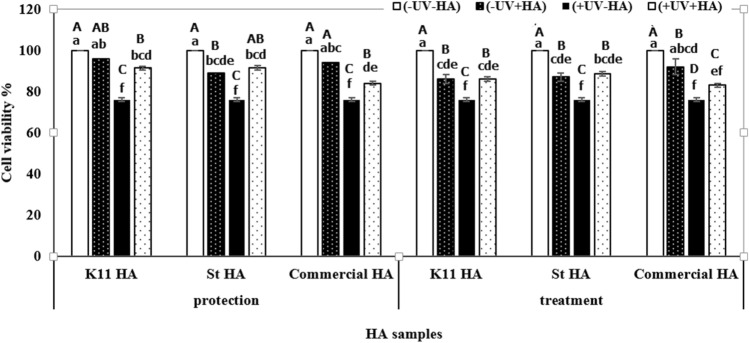
The viability of human dermal keratinocytes after exposure to (single dose 10 mJ/cm^2^) UVB radiation and pre- or post-treatment with HA produced by *E. durans* strain K11 and *L. plantarum* strain St3. Data are shown as the mean ± SE of triplicate measurements from independent experiments. Statistical significance was assessed using one-way ANOVA (analysis of variance, SPSS software v.18) test and the means were compared with Duncan’s test at 0.05 level. Accordingly, mean values with different small letters from different treatments of all tested HA samples, K11 HA, St HA, and commercial HA are considered statistically different (*P*≤0.05). While mean values with capital letters from different treatments at each tested HA sample, separately, are considered statistically different (*P* ≤ 0.05)

All untreated and non-irradiated human dermal keratinocytes showed the highest viability for pre- and post-treated cells. The results obtained from the pre- and post-treatment experiments after 24 h from the exposure of human dermal keratinocytes to the selected dose of UVB radiation (10 mJ/cm^2^) revealed that the viability of irradiated untreated cells significantly decreased (at *P*≤0.05). There is a significant difference (at *P*≤0.05) in the viability of UVB-irradiated cells pre-treated with HA extracted from K11 and St3 compared with that pre-treated with commercial HA. The results presented that HA extracted from K11 and St3 significantly maintained the viability of the cells by 91.3% and 91.4% in comparison to the absence of HA by 76%, respectively. Also, there is a significant difference (at *P*≤0.05) in the viability of UVB-irradiated cells post-treated with HA extracted from K11 and St3 compared with that post-treated with commercial HA. The results showed that HA extracted from K11 and St3 significantly maintained the viability of the cells by 86% and 88.5% in comparison to the absence of HA by 76%, respectively (Fig. 5).

## Discussion

Probiotics are valuable components that have been used for centuries and applied in many different industries, including food products, pharmaceuticals, and various health benefits. Collective number of studies indicate that bacterial substances such as cell wall fragments, their metabolites, and dead bacteria can trigger specific immune responses on the skin and enhance barrier function. Many studies have revealed the use of probiotic extracts for topical application on the skin [31].

In dermatology, hyaluronic acid (HA) is frequently used as a biomaterial for both wound healing stimulation and bioengineering applications. HA is frequently employed in ophthalmology, rheumatology, pharmacology, and drug delivery in addition to dermatological and cosmetic products [32].

In the present study, 18 probiotic isolates were isolated on MRS, kanamycin aesculin azide, and KF streptococcus agar media which are proper media used to isolate probiotic strains, from different three sources [17, 33]. In addition to *L. plantarum* strain M*, L. plantarum* strain St3*, L. casei* 1922, and *L. acidophilus* 23,431.

All studied isolates were screened for their ability to produce hyaluronic acid on skimmed milk medium. This is parallel to Izawa et al. [34] and Kanamarlapudi and Muddada [35] who isolated hyaluronic acid producing *S. thermophilus* on skimmed milk medium. Also, Lew et al. [11] reported that lactobacilli and bifidobacteria in skimmed milk were able to produce hyaluronic acid.

The concentration of the produced hyaluronic acid using CTAB turbidimetric method was assessed. This method is a quick, safe, and accurate method to determine HA concentration in fermentation culture compared with colorimetric carbazole method [36]. The amount of turbidity developed when CTAB is added to HA solution is proportional to the amount of HA in sample [37]. The results showed that fifteen out of twenty-two bacterial isolates were able to produce HA ranging from 0.651 to 1.316 mg/ml. This is parallel to the findings of Lew et al. [11] who reported that hyaluronic acid was produced in range of 0.2–1.4 mg/ml by certain lactobacilli and Bifidobacteria. While the results exceed the findings of Choi et al. [13] who demonstrated that hyaluronic acid production by lactobacilli strains grown in MRS broth for 20 h at 37 °C ranged from 0.236 to 0.303 mg/ml.

The hyaluronic acid produced by the selected isolates was quantitatively analyzed using HPLC analysis. The results of HPLC analysis revealed that the HA standard and all the produced HA had the same retention time and the HA concentrations produced by the selected isolates ranged from 0.2 to 4.8 mg/ml. Kakehi et al. [38] reported that HPLC is a very effective technique and can determine HA in the range 0.5–50 µg with high reproducibility. This is in accordance with Hamad et al. [23] who reported that HA concentration was 1.73 and 2.95 mg/ml by *S. thermophilus* TH-4 and *S. thermophilus* BLM 58, respectively. Also, Izawa et al*.* [34] and Mohammed and Niamah [39] confirmed HA production by *S. thermophilus* using HPLC technique.

From the previous studies, turbidimetric assay is less accurate in determining the hyaluronic acid concentration compared with that obtained by HPLC. Also, the HA-CTAB complex formation depends significantly on the ionic composition and strength. Therefore, the CTAB precipitation is applicable only for rough estimations of HA in certain samples, such as a microbial fermentation medium, whereas HPLC method is sensitive and specific [40]. This is in accordance with Hamad et al. [23] who reported that the productivity which analyzed with HPLC was increased with percentage 74 and 80% of that by turbidimetric assay.

The FTIR spectroscopy is an important technique to identify the functional groups and organic compounds by assessing the transitions between vibrational states of bonds contained within the molecule [23]. FTIR analysis is a qualitative technique and was carried out to confirm the identity of the produced HA comparing with the standard. Similarly, Zamboni et al. [41] and Mohammed and Niamah [39] analyzed and confirmed HA functional groups by FTIR.

Data obtained from CTAB turbidimetric assay and confirmed with HPLC revealed that the highest HA production was achieved by K11 and St3 isolates which were selected as the most potent HA producing probiotic isolates. The probiotic isolate St3 was identified using 16S rRNA gene sequence by Shaheen [20]*,* as *L. plantarum* strain St3 (KY508301), while the isolate K11 was identified using 16S rRNA gene sequence as* E. durans* strain K11 (ON359827). This is the first record of HA production by *E. durans* and *L. plantarum.* The production of hyaluronic acid using novel probiotic bacteria was a main objective of this study and our results revealed that it has been successfully attained. Previous studies reported that HA producing bacteria was achieved by pathogenic strains *S. zooepidemicus*. It was reported for the first time in 2009 that HA is produced in milk broth through fermentation by a putative probiotic strain *Streptococcus thermophilus* YIT2084 [35], while limited studies demonstrate the production of hyaluronic acid from lactic acid bacteria; *S. thermophiles, L. casei, L. fermentum, L. gasseri, L. acidophilus, L. bulgaricus, Bifidobacterium bifidum, and B. longum* which recorded as HA- producing bacteria [39]. Also, Fotouhi et al. [42] reported that Generally Recognized As Safe (GRAS) bacteria including *L. acidophilus* PTCC1643, *L. rhamnosus* PTCC1637, *L. casei* PTCC1608 and *S. thermophilus* PTCC1738 were able to produce HA**.**

Out of all the in vitro methods, DPPH scavenging method is the most easy, simple, and reasonably costly method for estimating the free radical-scavenging activities of antioxidants. The antioxidant activity of the HA produced by *Enterococcus durans* strain K11 and *L. plantarum* strain St3 were (65.4 ± 0.2%) and (66.6 ± 0.1%), respectively. This is in accordance with Pan et al. [43] who recorded that the highest scavenging effect was 41% for the produced HA. The mechanism by which HA reduces damage from free radicals is based on its structure, which has carboxylic groups responsible for its antiradical properties [43].

In the present study, the response of human dermal keratinocytes to UVB irradiation was in vitro evaluated. The viability of the irradiated untreated cells was 76% while the viability of pre-treated irradiated cells was 91.3 and 91.4% for HA produced by *Enterococcus durans* strain K11 and *L. plantarum* strain St3, respectively. Also, the viability of irradiated post-treated cells was 86 and 88.5% for HA produced by *Enterococcus durans* strain K11 and *L. plantarum* strain St3, respectively. This is in accordance with Hašová et al. [28] who recorded that HA produced by *Streptococcus equi* had significant protective effects for HaCaT keratinocytes against UVB irradiation.

## Conclusion

Data obtained from all experiments suggest three significant conclusions. First, *Enterococcus durans* strain K11 and *L. plantarum* strain St3, which belongs to probiotic bacteria, can produce exopolysaccharides, such as HA. Second, in an in vitro antioxidant assessment, the produced HA showed much higher antioxidant activity. Third, the application of HA could protect the human dermal keratinocytes before and after exposure to UVB radiation. HA could, therefore, be act as a protective agent that prevents the harmful effect of solar irradiation on skin.

## Supplementary Information

Below is the link to the electronic supplementary material.

Supplementary file1 (TIFF 665 KB) Figure (S1): HPLC analysis of the produced hyaluronic acid by the selected isolates A: standard HA, B: K2, C: K4, D: K11, E: K13, F: K33, G: K44, H: H3, I: H4, J: H5, K: Z3, L: Z4, M: M, N: St3, O: 1922, and P: 23431.

Supplementary file2 (TIFF 450 KB)

Supplementary file3 (TIFF 733 KB)

Supplementary file4 (TIFF 610 KB) Figure (S2): FTIR analysis of the produced hyaluronic acid by the selected isolates A: standard HA, B: K4, C: K11, D: K33, E: H3, F: H4, G: H5, H: St3, and I: 1922).

## Data Availability

The identified strains in this study have been deposited in NCBI database under the accession numbers KY508301 and ON359827.
