# Supplementary figures and images for: Photoprotection Against UV-Induced Skin Damage Using Hyaluronic Acid Produced by Lactiplantibacillus plantarum and Enterococcus durans
Source: Curr Microbiol. 2023 Jun 27;80(8):262. doi: 10.1007/s00284-023-03377-y (PMC10299924; doi:10.1007/s00284-023-03377-y)

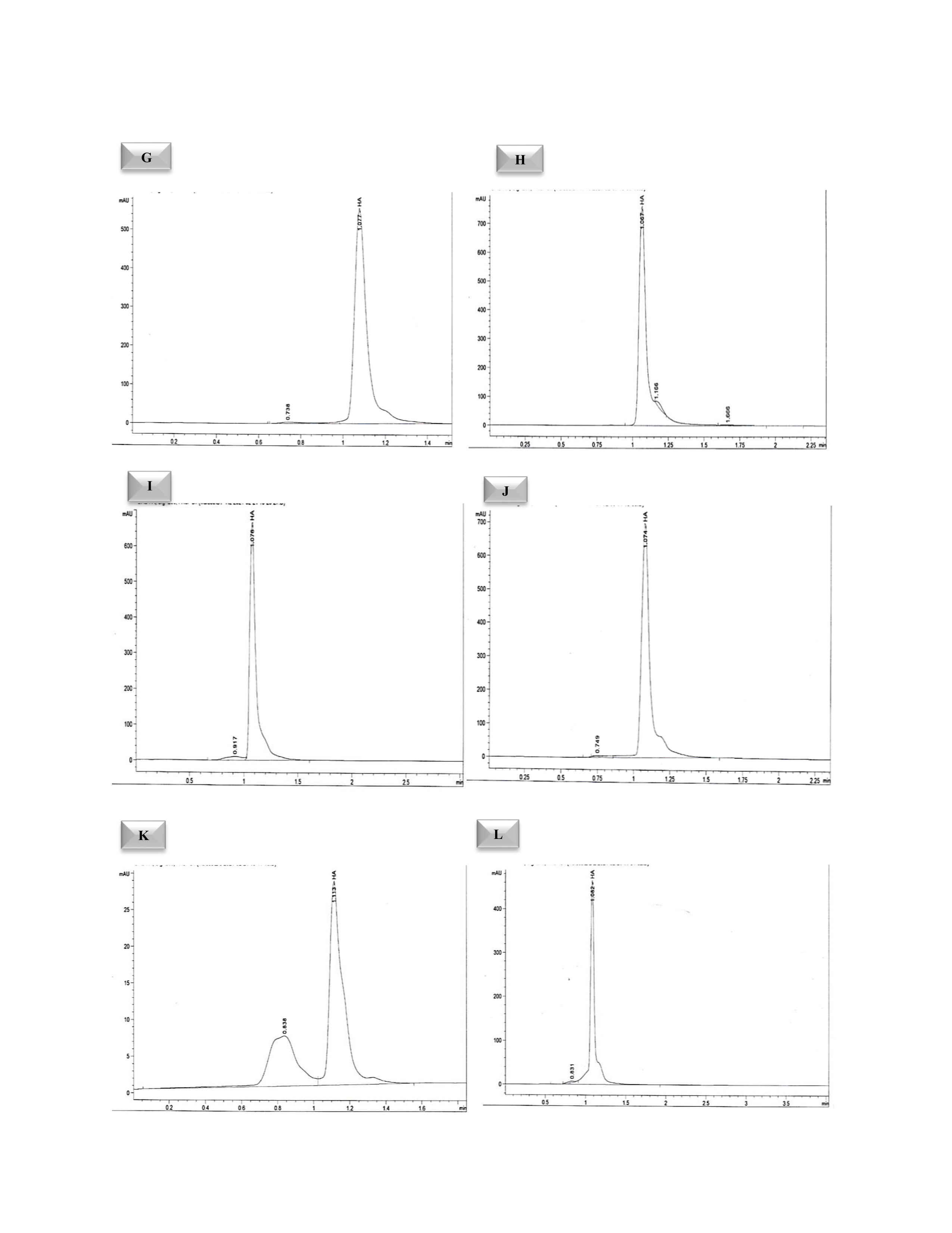

Supplement: Supplementary file 1 — Supplementary file1 (TIFF 665 KB) Figure (S1): HPLC analysis of the produced hyaluronic acid by the selected isolates A: standard HA, B: K2, C: K4, D: K11, E: K13, F: K33, G: K44, H: H3, I: H4, J: H5, K: Z3, L: Z4, M: M, N: St3, O: 1922, and P: 23431. [file 284_2023_3377_MOESM1_ESM.tiff]

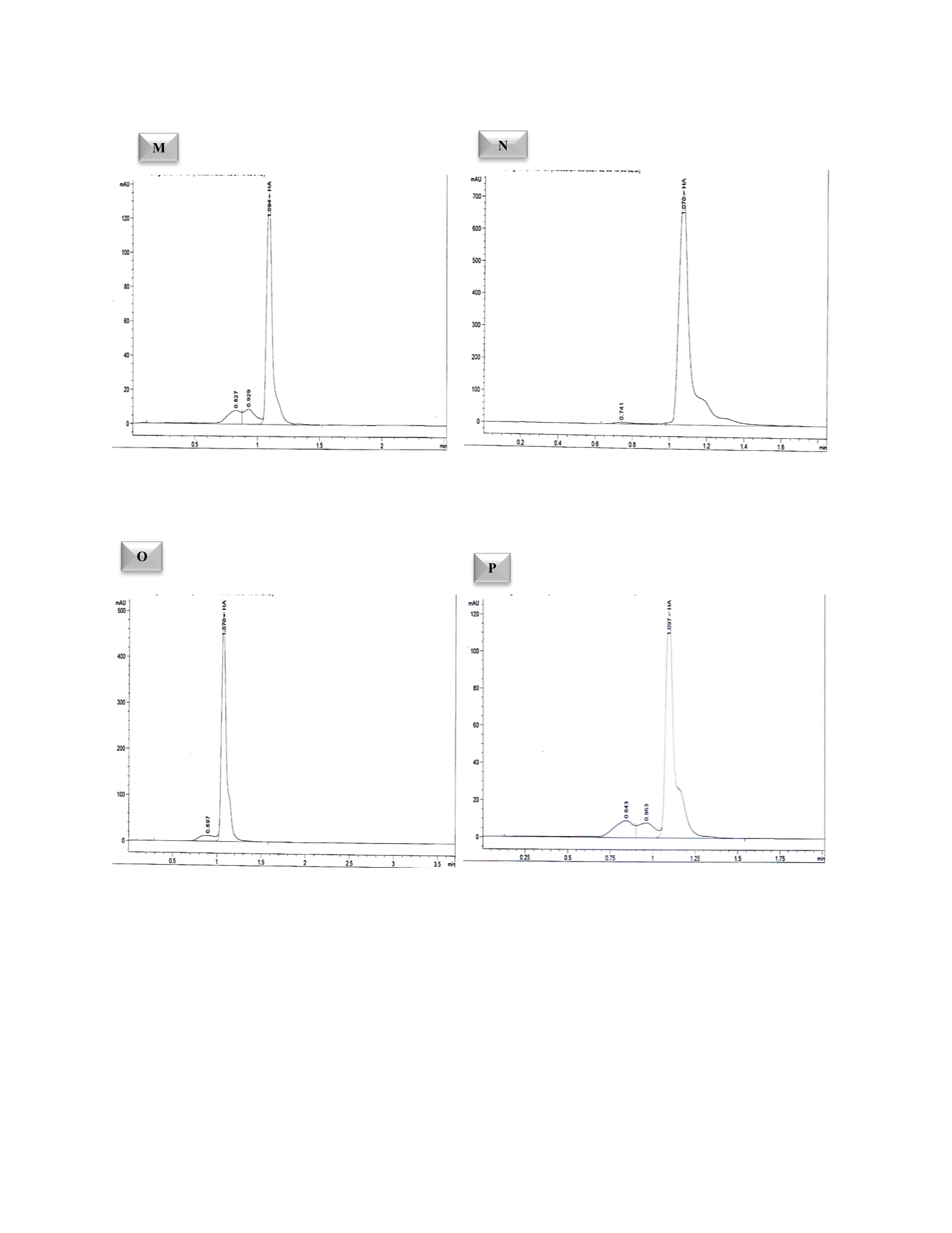

Supplement: Supplementary file 2 — Supplementary file2 (TIFF 450 KB) [file 284_2023_3377_MOESM2_ESM.tiff]

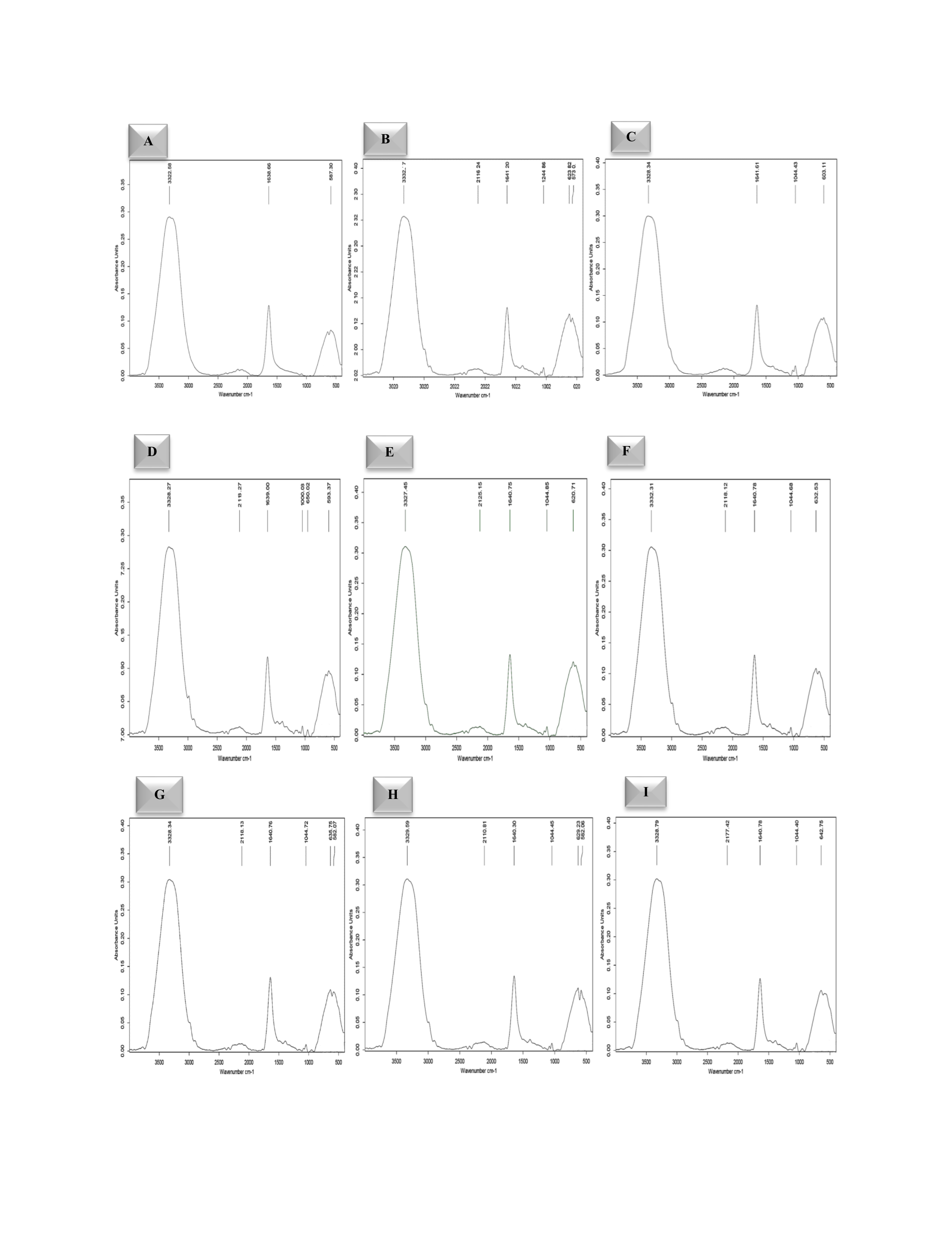

Supplement: Supplementary file 3 — Supplementary file3 (TIFF 733 KB) [file 284_2023_3377_MOESM3_ESM.tiff]

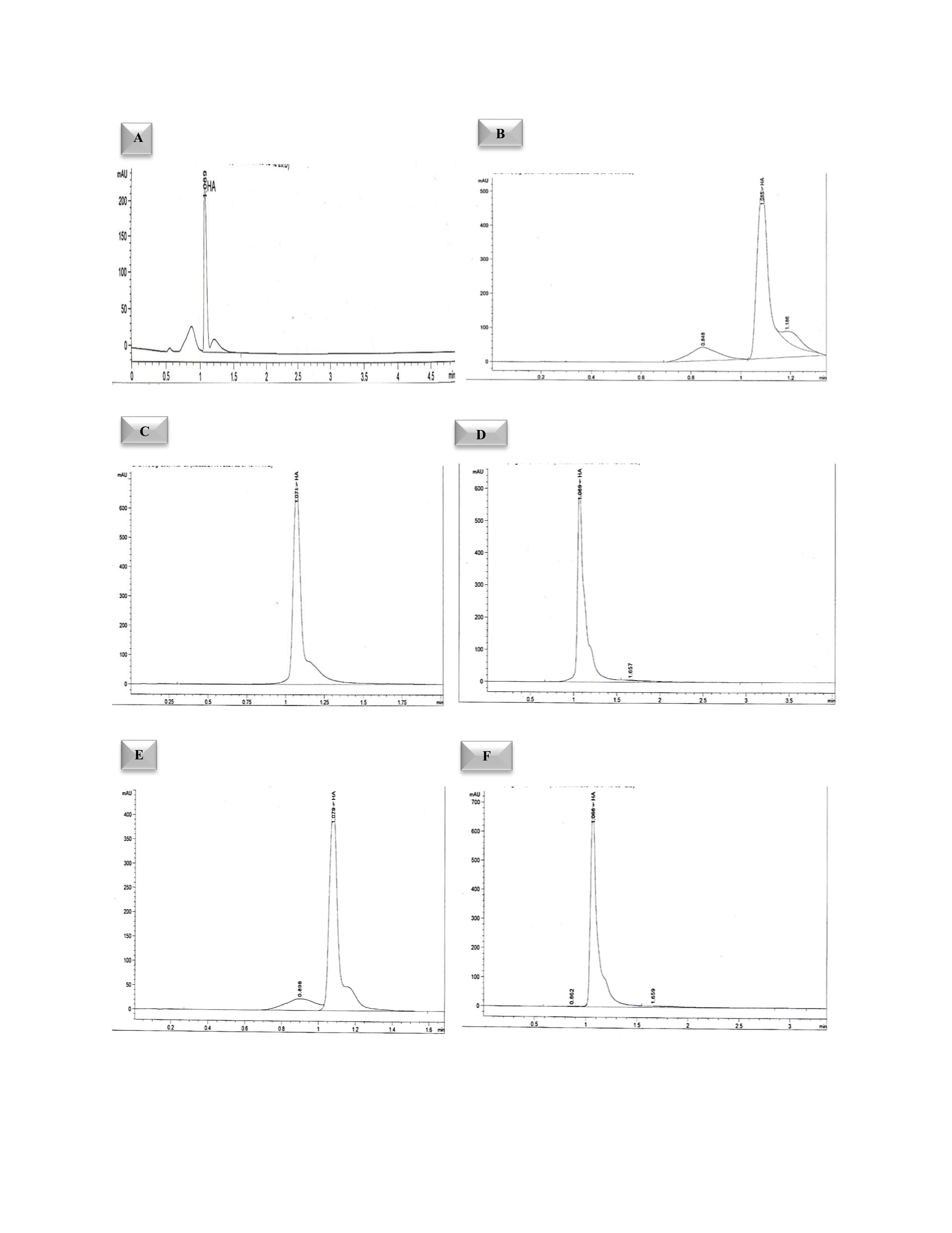

Supplement: Supplementary file 4 — Supplementary file4 (TIFF 610 KB) Figure (S2): FTIR analysis of the produced hyaluronic acid by the selected isolates A: standard HA, B: K4, C: K11, D: K33, E: H3, F: H4, G: H5, H: St3, and I: 1922). [file 284_2023_3377_MOESM4_ESM.tiff]
